# Alterations of Dopamine Receptors and the Adaptive Changes of L-Type Calcium Channel Subtypes Regulate Cocaine-Seeking Habit in Tree Shrew

**DOI:** 10.3390/life12070984

**Published:** 2022-06-30

**Authors:** Ying Duan, Lingtong Jin, Wenjie Du, Shubo Jin, Yiming Meng, Yonghui Li, Jianjun Zhang, Jing Liang, Nan Sui, Fang Shen

**Affiliations:** 1 Key Laboratory of Mental Health, Institute of Psychology, Chinese Academy of Sciences, Beijing 100101, China; duanyingpsy@gmail.com (Y.D.); jinlt@psych.ac.cn (L.J.); w.du-2@umcutrecht.nl (W.D.); shuboj@student.unimelb.edu.au (S.J.); mengym2021@ion.ac.cn (Y.M.); liyonghui@psych.ac.cn (Y.L.); zhangjj@psych.ac.cn (J.Z.); liangj@psych.ac.cn (J.L.); suin@psych.ac.cn (N.S.); 2 Department of Psychology, University of Chinese Academy of Sciences, Beijing 100101, China

**Keywords:** cocaine-seeking habit, putamen, DA receptors, LTCCs, tree shrew

## Abstract

The putamen (Put) is necessary for habitual actions, while the nucleus caudate (Cd) is critical for goal-directed actions. However, compared with the natural reward (such as sucrose)-seeking habit, how drug-related dysfunction or imbalance between the Put and Cd is involved in cocaine-seeking habit, which is not easy to bias behavior to goal-directed actions, is absent. Therefore, in our present study, in comparison with sucrose-habitual behavior, we evaluated the distinctive changes of the two subtypes of dopamine (DA) receptors (D1R and D2R) in cocaine-seeking habitual behavior animals. Moreover, the adaptive changes of Ca_v_1.2 and Ca_v_1.3, as prime downstream targets of D1R and D2R respectively, were also assessed. Our results showed that a similar percentage of the animals exhibited habitual seeking behavior after cocaine or sucrose variable-interval self-administration (SA) training in tree shrews. In addition, compared with animals with non-habitual behavior, animals with cocaine habitual behavior showed higher D1Rs and Ca_v_1.2 expression in the Put accompanied with lower D2Rs and Ca_v_1.3 expression in the Cd. However, after sucrose SA training, animals with habitual behavior only showed lower membrane expression of D2R in the Put than animals with non-habitual behavior. These results suggested that the upregulation of D1Rs-Ca_v_1.2 signaling may lead to hyper-excitability of the Put, and the inactivation of D2Rs-Ca_v_1.3 signaling may result in depressed activity in the Cd. This imbalance function between the Put and Cd, which causes an inability to shift between habits and goal-directed actions, may underlie the compulsive addiction habit.

## 1. Introduction

Although behaviors typically are dominated by the explicit representation of desired outcomes, some behaviors, called habits, seem to struggle against these conscious bonds. Intuitively, habits, an efficient mode of information processing, serve an obvious adaptive purpose. They are usually triggered by certain stimulus automatically, which are associated with the completion of the previous behavior and the presentation of the outcome (stimulus–response association, S-R). However, habits also tend to be inflexible in some circumstances even when the environment changes. This tendency is amplified in substance abuse, which has been taken as one of the most important underlying factors for drug craving and relapse. Many studies have found that, not like habits in life, drug-seeking habits are inclined to be compulsive [1]. Indeed, in humans, when alcohol-dependent people conducted a task that can distinguish between goal-directed (A-O) or S-R strategy, they tended to overuse the S–R association [2]. In rodents, after long-term noncontingent exposure to addictive drugs, animals also tended to overly depend on the habit system despite adverse consequence [3,4]. However, how drug-related dysfunction or imbalance between the A-O strategy and S-R strategy involved in cocaine-seeking habit is absent.

It has been deeply understood that there are functional alterations from the nucleus caudate (Cd) to the putamen (Put) during the development of substance abuse. The Put mainly participates in regulating habitual behaviors, while the Cd plays a role in mediating goal-directed actions [5]. Various evidence pointed to the functional unbalance between Cd and Put may underlie compulsive drug-seeking habits. Indeed, fMRI studies have found that the Put represented overactive states specific in alcohol-dependent participants [6], suggesting that the hyperactive Put might be the main factor causing compulsive elements of drug-seeking habits.

Within the striatum, the predominant cell types are GABAergic medium spine neurons (MSNs), which are typically segregated into dopamine 1 receptor (D1R) or dopamine 2 receptor (D2R) containing [7]. These cells have different projection targets and serve distinctive functions in the reward processes. This functional discrepancy is partly because D1Rs and D2Rs have different biological characteristics [8]. They have different downstream molecular targets, dopamine affinity, and represent functional antagonists, modulating neural activity differently [9]. For instance, D1Rs and D2Rs signaling participate in plasticity at glutamatergic synapses respectively: long-term potentiation (LTP) in the striatum depends on the action of D1Rs, whereas long-term depression (LTD) in the striatum relies upon that of D2Rs [8,10]. More importantly, the different changes of these subtype receptors may underlie the compulsive elements of cocaine-seeking habitual behavior via mediating neural activity or even plasticity. Indeed, studies have shown that during cocaine habitual behavior under a second-order schedule of reinforcement, dopamine overflow increased in the Put, and infusion of a non-selective dopamine receptor antagonist into the Put reduced cocaine-seeking habits [11,12]. Although many studies have focused on dopamine signaling in habitual behaviors, few know how D1Rs and D2Rs work differently [13]. Therefore, we hypothesized that plasticity changes in the dorsal striatum subregions, which are modulated by D1Rs and D2Rs, which probably lead to the hyperactivity of Put, could be the key mechanism of compulsive elements of cocaine-seeking habits.

As one of the downstream targets of DA receptors, L-type calcium channels (LTCCs) are essential for the neuronal in the striatum [14,15]. Notably, as downstream agents of DA receptors, Ca_v_1.2 and Ca_v_1.3, the two most prominent LTCCs subtypes distributed in the brain, are regulated by D1Rs and D2Rs, respectively [16]. In comparison with Ca_v_1.2, Ca_v_1.3 channels are activated more rapidly and at more negative membrane potentials [16]. Additionally, the activation of Ca_v_1.2 via D1Rs is the key phrase during the formation of LTP, whereas the activation of Ca_v_1.3 via D2Rs is the main form of LTD in the striatopallidal neurons [10]. Based on the above evidence, we hypothesized that the D1Rs-Ca_v_1.2 signaling and the D2Rs-Ca_v_1.3 signaling might participate distinctively in sucrose-seeking and cocaine-seeking habits.

Tree shrews are increasingly being used as a new and promising animal model in neurobiological studies [17,18]. Compared with rodents, they are genetically closer to the primate [19], and, notably, have a clearer anatomical structure in the striatum to distinguish between Cd and Put [20]. Moreover, in our lab, tree shrews have been proved also suitable to establish addiction models [21]. Therefore, in the present study, using tree shrews, we explored the differential expression of D1Rs and D2Rs in the Put and Cd in habitual behavior established on natural rewards and cocaine, respectively, which is necessary for understanding the compulsive characteristics of drug addiction.

## 2. Methods and Materials

### 2.1. Animals

Adult male tree shrews (Tupaia belangeri chinensis; 130–160 g) were used (the Animal House Center of the Kunming Institute of Zoology). All animals were individually housed in rearing cages (395 × 300 × 595 mm), each of which was attached to a nest box (246 × 158 × 147 mm) that can provide sleeping quarters and functioned as a transfer box when the animal was moved from its home cage to the training apparatus. The tree shrews were kept in an air-conditioned room in which the temperature (22–25 °C) and the humidity (40%–70%) were controlled on a 12 h/12 h dark/light cycle (lights on at 8:00) for at least two weeks prior to experiments, which ensured the tree shrews adapted to the new environment. Water and food (purchase from Keaoxieli Co., Beijing, China) were available ad libitum. All procedures were conducted according to the National Institutes of Health Guide for the Care and Use of Laboratory Animals and were approved by the Research Ethics Review Board of the Institute of Psychology, Chinese Academy of Sciences (A22039).

### 2.2. Drug Administration

Cocaine hydrochloride (Qinghai Pharmaceutical, Qinghai, China) was dissolved in 0.9% sterile physiological saline to the final concentrations.

### 2.3. SA Apparatus

The tree shrews were trained and tested for self-administration (SA) in standard operant chambers for rats (Med Associates, Inc., St. Albans, VT, USA), which were placed in a sound insulation cubicle. Each chamber was installed with two cue lights in two nose-poking holes (ENV-114M, Med Associates) situated 2 cm above the floor which were equipped with horizontal bars and a house light was located on the opposite wall. The drug solution was delivered through polyethylene tubing, protected by a leash assembly (PHM-120, Med Associates), and the polyethylene tubing connected with a fluid rotary joint (PHM-115, Med Associates), which was poised through the ceiling of the chamber. The drug solution was delivered by a 10 mL syringe in an infusion pump (PHM-100, Med Associates). Lenovo computer with MED PC Software IV (Med Associates) controlled infusions and light presentations and recorded the number of nose pokes.

### 2.4. Surgery

In the cocaine group, tree shrews were anesthetized with pentobarbital sodium (100 mg/kg, i.p.) to implant an intravenous jugular catheter (AniLab, Ningbo, China). A silicone tube was inserted 35 mm into the right jugular vein and firmly anchored to the vein with a silk suture. The other end of the catheter passed subcutaneously to connect with a 22-gauge connector (Plastics One, Roanoke, VA, USA) mounted on the back and was plugged with a solid pin when it was not being used for drug infusions.

### 2.5. Western Blot Analysis

For the western blot experiments, tree shrews were decapitated at once after the devaluation test and the brains were removed and frozen immediately in N-hexane (−70 °C) for approximately 30 s. Bilateral tissue punches of the Put and the Cd were obtained by using a 16-gauge needle in cryostat (Leica). The details of the extraction of protein were described previously [16]. Briefly, an equal amount of protein (30 ug for total protein and 20 ug for membrane protein) for each sample was resolved on 8% sodium dodecyl sulphate–polyacrylamide gel electrophoresis (SDS-PAGE) gels and transferred to polyvinylidene difluoride (PVDF) membranes (Millipore, Burlington, MA, USA). Then, membranes were incubated in Tris-buffered saline (TBS) (50 mM Tris-HCl, pH 7.4, 150 mM NaCl, and 0.05% Tween 20) with 5% nonfat dry milk for 2 h at room temperature, and incubated with the following primary antibodies overnight at 4 °C: anti-Ca_v_1.2 (Alomone Lab, Jerusalem, Israel), anti-Ca_v_1.3 (Alomone Lab, Jerusalem, Israel), anti-D1R (Santa Cruz Biotechnology, Santa Cruz, CA, USA), anti-D2R (Abcam, Cambridge, MA, USA), and anti-β-actin Sigma, St. Louis, MO, USA). Membranes were washed in TBS/0.1% Tween 20 three times for 5 min and were then incubated in an anti-rabbit or anti-mouse secondary antibody conjugated to horseradish peroxidase (Zhongshan Biotechnology, Zhongshan City, Guangdong, China). Finally, the membranes were developed using West Dura chemiluminescent substrate (Pierce Laboratories, Waltham, MA, USA). The bands were quantified with Quantity One Analysis Software (Bio-Rad, Hercules, CA, USA). The optical density of each band was normalized to the relative optical density of β-actin protein expression to control for inconsistencies between the loaded samples.

### 2.6. Behavioral Procedures

Cocaine SA training sessions began 7 days following surgery. Cocaine (0.175 mg/0.05 mL per infusion in 2.5 s, intravenous injection) or 10% sucrose solution (0.2 mL per drop in 0.2 s, oral administration) was available under a fixed-ratio (FR) schedule and variable-interval (VI) schedule. The concentrations of cocaine and sucrose referred to the previous studies in our lab (please insert the references list in the comment). Three stages of both cocaine SA training and sucrose SA training were included in our study as previously described (Furlong et al., 2014), including FR1 training, VI training, and devaluation test. The details are described as follows.

**FR1 training:** Poking in the active hole resulted in an infusion and initiated a 20 s time-out. At the same time, the active hole-specific cue light was lighted as a conditioned stimulus (CS). The house light was turned off during the time-out. Poking in the inactive hole was recorded but had no consequence. The maximum infusions in cocaine SA training or sucrose SA training were 60 per session. Each of the FR1 sessions lasted 120 min or until the tree shrew reached maximum infusions. In the cocaine group, the animals received 8 sessions of FR1 training, while the sucrose group received 5 sessions of FR1 training. If animals could not meet the criterion that the variation of the number of active nose pokes within the last 3 sessions fell below 20%, they were excluded. Inactive and active nose poking assignments were counter balanced.

**VI training:** The VI training was introduced following FR1 training, in which the reward (cocaine or sucrose) would be delivered in variable intervals after poking the active hole. In the cocaine group, animals received VI training for 6 days, in which the average of the interval was from 5 s to 40 s. In the sucrose SA training, tree shrews received VI30s training for 5 continuous days. Each session lasted for two hours.

**Devaluation test:** All animals that finished the above two stages were able to receive a devaluation test. The devaluation test occurred in the same conditions as for FR1 sessions but without rewards. The test lasted for 40 min in the cocaine group and 60 min in the sucrose group. The number of nose pokes was recorded every 10 min. Through this test, tree shrews could be divided into habitual and non-habitual groups. The habitual group maintained the number of nose pokes during the last three ten-minute intervals, while the non-habitual group rapidly decreased the number of nose pokes.

### 2.7. Statistical Analysis

For behavioral experiment, data were analyzed using two-way ANOVA, followed by the Bonferroni *post hoc* tests. For the western blot analysis, normalized optical density values were used to calculate the percentage fold change for each treatment group compared with the naïve tree shrews (set to 1), and these data were analyzed with *t*-test. All data were shown as the mean ± SEM and were processed in Graph Pad Prism 7.0.

## 3. Results

**The establishment of habitual cocaine-seeking behaviors in tree shrews.**

**Figure 1:**
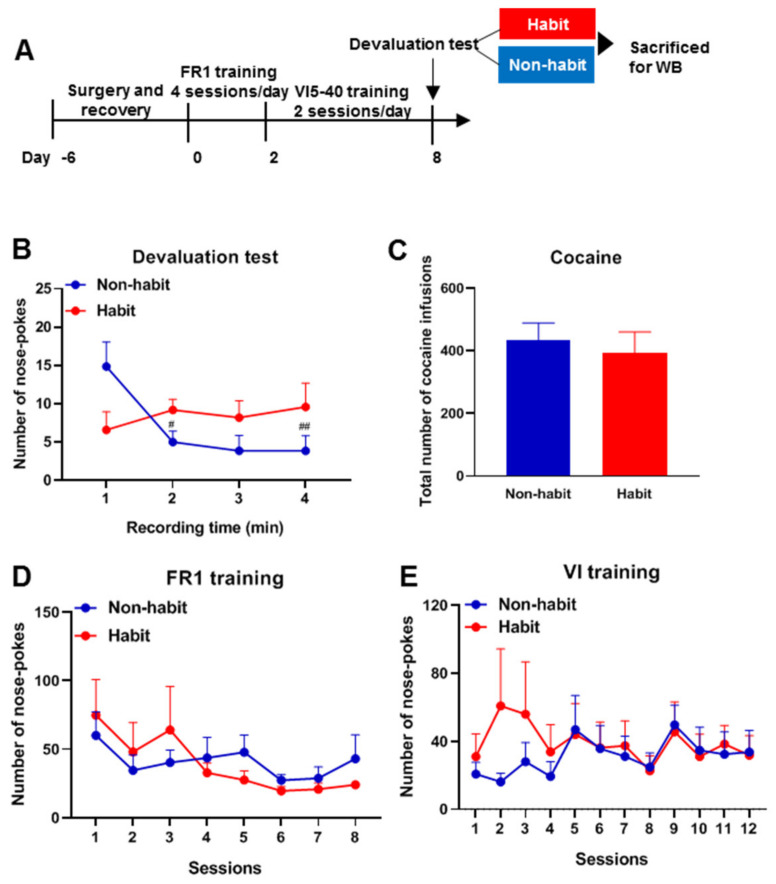
**The establishment of habitual cocaine-seeking behavior in tree shrews.** (**A**) The protocol for the training of cocaine habitual behavior. (**B**) In the devaluation test after variable interval (VI) training, the habit group exhibited a slightly increase nose pokes during each 10-min interval compared with the first 10 min, which is also not high enough to be statistically significant, but the non-habit group exhibited a decreased number of nose pokes in the periods of 10–20, 20–30, and 30–40 min compared with the period of 0–10 min. ^#^ *p* < 0.05, ^##^ *p* < 0.01, the number of valid nose pokes in the non-habit group compared with the number of valid nose pokes in the first 10 min. (**C**) The received dose of cocaine was no different between the non-habit tree shrews and the habit tree shrews. (**D**) The number of valid nose pokes in the FR1 training was no different between the two groups. (**E**) The number of valid nose pokes in the VI training was no different between the two groups. The data were expressed as the means ± SEM and analyzed with a two-way ANOVA followed by the Bonferroni post hoc test or the *t*-test, habit group *n* = 5, non-habit group *n* = 7.

In past research, paradigms to examine the isolated goal-directed and habitual actions have been developed and outcome devaluation procedures are commonly used to detect whether a behavior is controlled by a goal or a habit [22,23]. The devaluation test was conducted after the VI training phase in our research (Figure 1A). After the entire training in our study, we found that tree shrews demonstrated two opposite trends in seeking behaviors (Two-way ANOVA, group × time effect: F_(3, 30)_ = 4.657; *p* = 0.009) (Figure 1B). The number of valid nose pokes in some tree shrews remained at a stable level in the devaluation test (One-way ANOVA, F_(3, 16)_ = 0.3263, *p* = 0.8063; *n* = 5), which means that the number of the valid nose pokes during the last three ten-minute intervals did not exceed 20% of the decreases from the first 10 min [24], indicating that this group of tree shrews performed habitual behaviors that were insensitive to devaluation; however, the number of valid nose pokes in other tree shrews decreased significantly (One-way ANOVA, F_(3, 24)_ = 5.625, *p* = 0.0046, *n* = 7), indicating that they performed goal-directed behaviors. However, no difference was observed in the received dose of cocaine between the two groups (*t*-test, *t*_(10)_ = 0.475, *p* = 0.645) (Figure 1C), and no differences were observed in the number of valid nose pokes during the entire FR1 (Two-way ANOVA, group effect: F_(1, 10)_ = 0.0229, *p* = 0.883; session effect: F_(3.089, 30.89)_ = 2.494, *p* = 0.0768; interaction effect: F_(7, 70)_ = 0.837, *p* = 0.561) and VI training process (Two-way ANOVA, group effect: F_(1, 10)_ = 0.321, *p* = 0.583; session effect: F_(2.457, 24.57)_ = 0.849, *p* = 0.461; interaction effect: F_(11, 110)_ = 0.837, *p* = 0.603) (Figure 1D,E). 

These results showed that some tree shrews established cocaine-seeking habits after FR1 and VI trainings. Moreover, these results are independent of the consumption of cocaine and the magnitude of trainings.

**The protein levels of D1R and Ca_v_1.2 increased, whereas the protein levels of D2R and Ca_v_1.3 decreased in the Put of cocaine-habitual tree shrews.**

**Figure 2:**
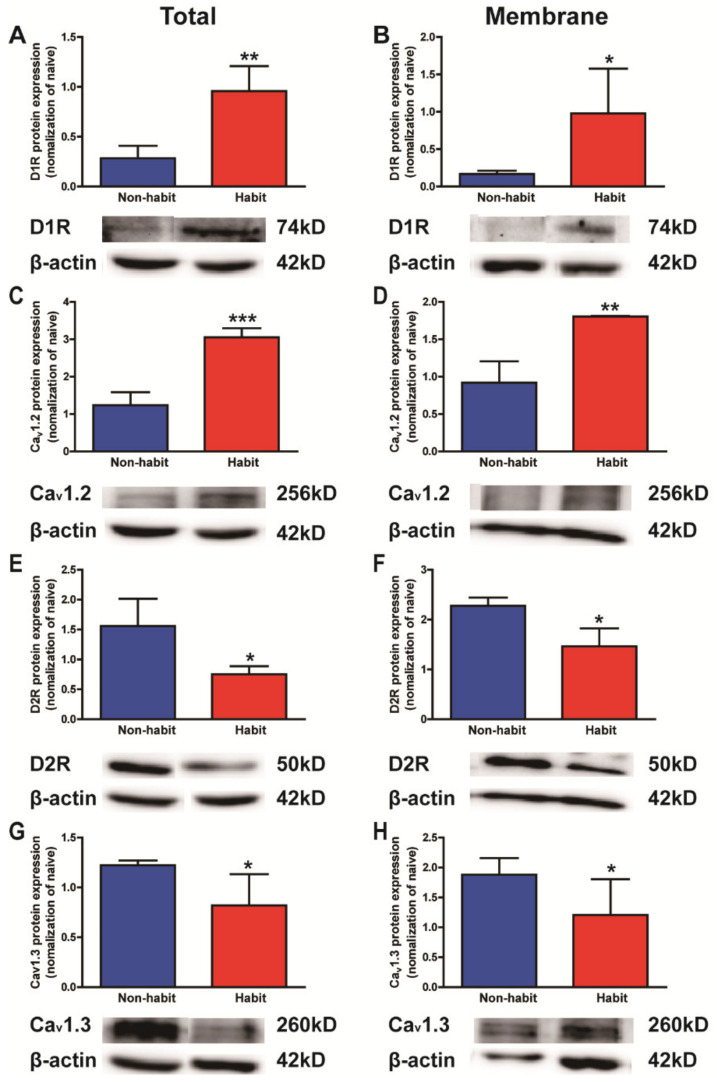
**The total and membrane protein levels of D1Rs vs. D2Rs, Ca_v_1.2 vs. Ca_v_1.3 in the Put.** (**A**) The total protein level of D1Rs in the Put was higher in the habit tree shrews than in the non-habit animals. (**B**) The membrane protein level of D1Rs in the Put was higher in the habit tree shrews than in the non-habit animals. (**C**) The total protein level of Ca_v_1.2 in the Put was higher in the habit tree shrews than in the non-habit animals. (**D**) The membrane protein level of Ca_v_1.2 in the Put was higher in the habit tree shrews than in the non-habit animals. (**E**) The total protein level of D2Rs in the Put was lower in the habit tree shrews than in the non-habit animals. (**F**) The membrane protein level of D2Rs in the Put was lower in the habit tree shrews than in the non-habit animals. (**G**) The total protein level of Ca_v_1.3 in the Put was lower in the habit tree shrews than in the non-habit animals. (**H**) The membrane protein level of Ca_v_1.3 in the Put was lower in the habit tree shrews than in the non-habit animals. * *p* < 0.05, ** *p* < 0.01, *** *p* < 0.001; habit group vs. non-habit group. The data were expressed as the means ± SEM and analyzed with *t*-test, habit group *n* = 3, non-habit group *n* = 4.

After the devaluation test, all tree shrews were sacrificed and the protein levels of D1Rs, D2Rs, Ca_v_1.2, and Ca_v_1.3 in the Put were determined in both the habit and non-habit behavior groups of animals (Figure 2).

The statistical data indicated that both the total and membrane protein levels of D1Rs and Ca_v_1.2 in the Put were significantly higher in the habit tree shrews than in the non-habit animals (*t*-test, D1Rs total: *t* = 4.731, *p* = 0.0026; D1Rs membrane: *t* = 2.780, *p* = 0.0195; Ca_v_1.2 total: *t* = 7.626, *p* = 0.0003; Ca_v_1.2 membrane: *t* = 4.142, *p* = 0.0072) (Figure 2A–D), while both the total and membrane protein levels of D2Rs and Ca_v_1.3 in the Put were lower in the habit tree shrews than in the non-habit animals (*t*-test, D2Rs total: *t* = 2.938, *p* = 0.0212; D2Rs membrane: *t* = 3.556, *p* = 0.0118; Ca_v_1.3 total: *t* = 2.214, *p* = 0.0456; Ca_v_1.3 membrane: *t* = 2.025, *p* = 0.0494;) (Figure 2E–H).

Our results showed that the protein levels of D1Rs and Ca_v_1.2 increased, whereas the protein levels of D2Rs and Ca_v_1.3 decreased in the Put of the well-established cocaine habit tree shrews compared with the non-habit animals. 

**The protein levels of D1R, D2R, Ca_v_1.2, and Ca_v_1.3 had no difference in the Cd of cocaine-habitual tree shrews.**

**Figure 3:**
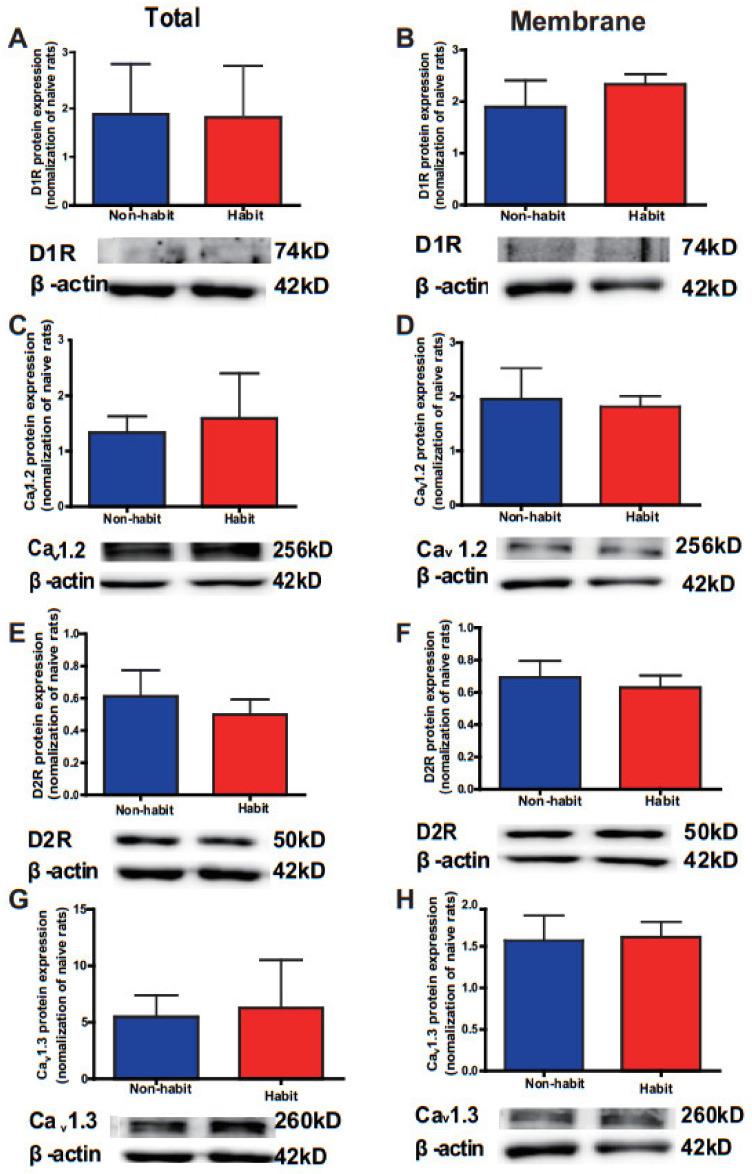
**The total and membrane protein levels of D1Rs vs. D2Rs, Ca_v_1.2 vs. Ca_v_1.3 in the Cd.** (**A**,**C**,**E**,**G**) No differences were observed in the total protein level of D1Rs, D2Rs, Ca_v_1.2, and Ca_v_1.3 in the Cd between the habit and the non-habit tree shrews. (**B**,**D**,**F**,**H**) No differences were observed in the membrane protein level of D1Rs, D2Rs, Ca_v_1.2, and Ca_v_1.3 in the Cd between the habit and the non-habit tree shrews. The data were expressed as the means ± SEM and analyzed with the *t*-test, habit group *n* = 3, non-habit group *n* = 4.

Meanwhile, we also detected the total and membrane protein levels of Ca_v_1.2, Ca_v_1.3, D1Rs, and D2Rs in the Cd in the habit group and non-habit group with the method of western blot. We observed no difference between the habit tree shrews and the non-habit animals in both total and membrane protein levels of these molecules in the Cd (Figure 3). Our results showed that the protein levels of Ca_v_1.2, Ca_v_1.3, D1Rs, and D2Rs in the Cd were no different between these two groups.

**The establishment of habitual sucrose-seeking behavior in tree shrews** **.**

**Figure 4:**
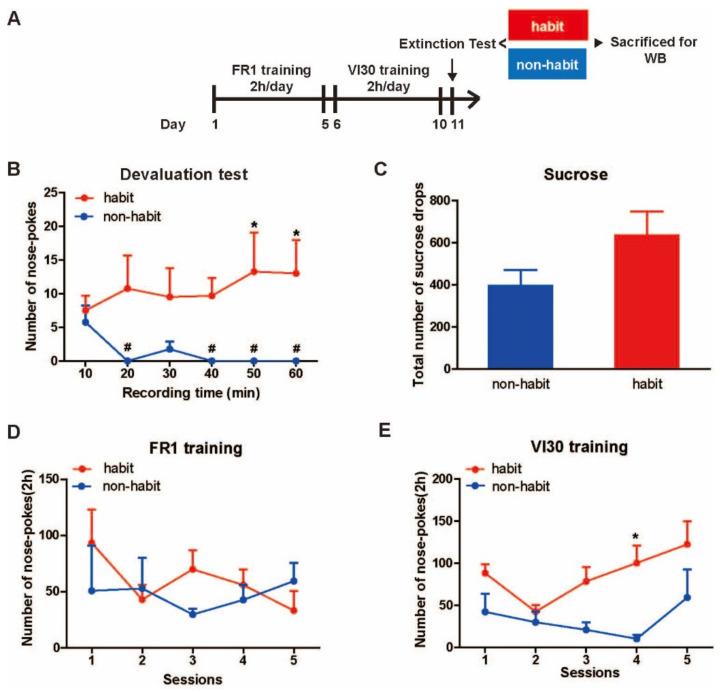
**The establishment of habitual sucrose-seeking behavior in tree shrews.** (**A**) The protocol for the training of sucrose habitual behavior. (**B**) In the devaluation test after VI training, the habit group exhibited no significant difference during the following five 10 min intervals compared with the first 10 min, while the non-habit group exhibited a decreased number of nose pokes in the periods of 10–20, 20–30, 30–40, 40–50, and 50–60 min compared with the period of 0–10 min. ^#^ *p* < 0.05 the number of valid nose pokes in the non-habit group compared with the number of valid nose pokes in the first 10 min; * *p* < 0.05,, the number of valid nose pokes in the non-habit group compared with the number of valid nose pokes in the habit group. (**C**) The received drops of sucrose was no different between the habit tree shrews and the non-habit tree shrews. (**D**) The number of valid nose pokes in the FR1 training was no different between the two groups. (**E**) The number of valid nose pokes in the VI training in the non-habit group was fewer than that in the habit group on the 1st, 3rd, and 4th sessions. * *p* < 0.05, habit vs. non-habit. The data were expressed as the means ± SEM and analyzed with a two-way ANOVA followed by the Bonferroni post hoc test or LSD post hoc test or the *t*-test, habit group *n* = 4, non-habit group *n* = 4.

The same as the cocaine SA training, after the entire sucrose SA training, tree shrews demonstrated two opposite directions in seeking behaviors (Figure 4A). The number of nose pokes in some tree shrews remained at a stable level (One-way ANOVA, *n* = 4; F _(3)_ = 0.758, *p* = 0.592), indicating that this group of tree shrews performed habitual behaviors that were insensitive to devaluation, while the number of nose pokes in other tree shrews decreased (One-way ANOVA followed by LSD *post hoc* test, *n* = 4, *p* < 0.001, 0.001, 0.001, 0.001, 0.001; 10–20, 20–30, 30–40, 40–50, 50–60 min vs. 0–10 min), indicating that they performed goal-directed behaviors. Moreover, two-way ANOVA revealed a significant difference within the group effect (F_(1, 36)_ = 14.175, *p* = 0.009) but no time effect (F_(5, 36)_ = 0.468, *p* = 0.797), nor interaction effect (F_(5, 36)_ = 1.480, *p* = 0.226). *t*-test showed that the number of nose pokes in the non-habit group at 10–20, 30–40, 40–50, and 50–60 min was significantly fewer than the habit group (*t* =3.797, *p* = 0.009; *t* = 3.151, *p* = 0.02; *t* = 2.997, *p* = 0.024; *t* = 3.205, *p* = 0.049). 

In addition, no difference was observed in the received rewards of sucrose between the two groups (*t*-test, *n* = 4, *t* = 2.282, *p* = 0.063) (Figure 4C), and no differences were observed in the number of nose pokes during the entire FR1 training (Two-way ANOVA, group effect: F_(1, 27)_ = 0.212, *p* = 0.669; session effect: F_(4, 27)_ = 0.327, *p* = 0.856; interaction effect: F_(4, 27)_ = 0.393, *p* = 0.811) (Figure 4D). Interestingly, during the VI training, two-way ANOVA revealed a significant difference within the group effect (F_(4, 28)_ = 17.1963, *p* = 0.008) and the number of valid pokes in the non-habit group was fewer than that in the habit group on the 1st, 3rd, and 4th sessions of training (*t*-test; *t* = 2.726, *p* = 0.034; *t* =3.753, *p* = 0.009; *t* = 6.310). These results showed that some tree shrews developed sucrose-seeking habits after VI trainings. 

**The membrane protein levels of D2R protein levels decreased in the Put of sucrose-habitual tree shrews.**

**Figure 5:**
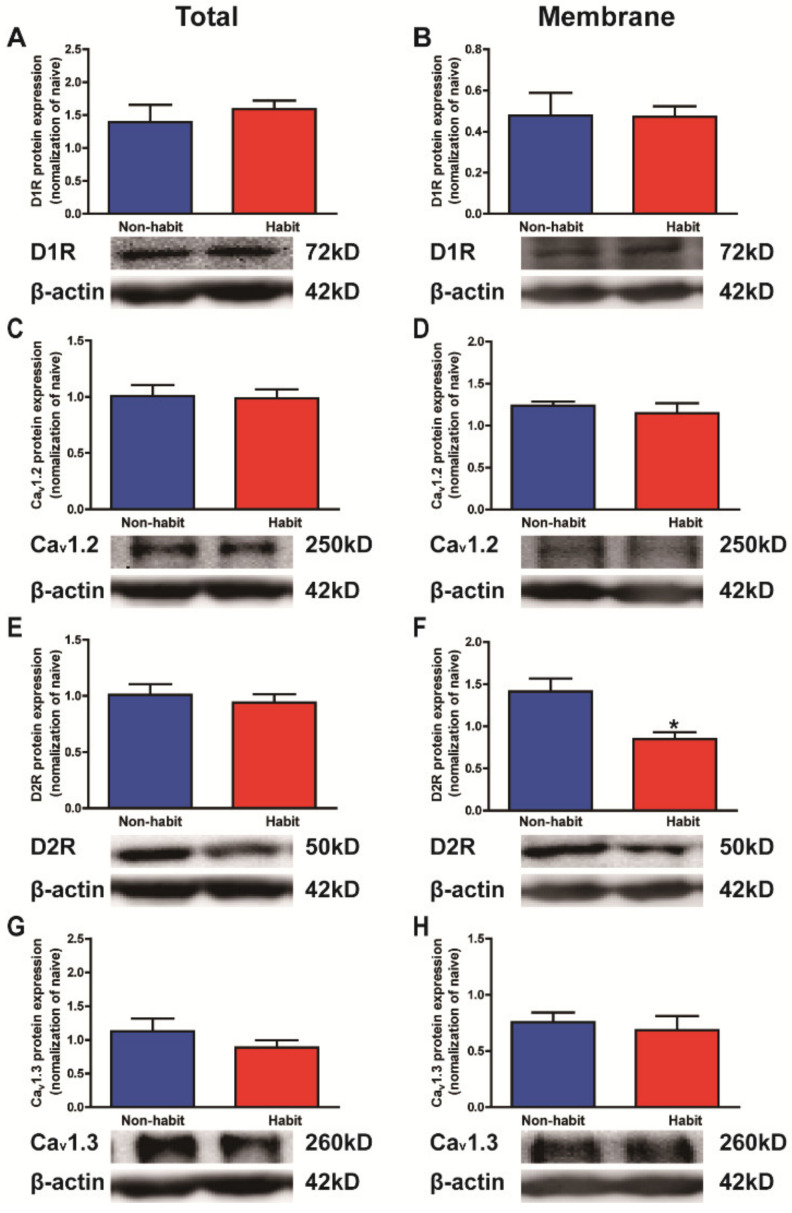
**The total and membrane protein levels of D1Rs vs. D2Rs, Ca_v_1.2 vs. Ca_v_1.3 in the Put.** (**A**,**C**,**E**,**G**) No differences were observed in the total protein level of D1Rs, D2Rs, Ca_v_1.2, and Ca_v_1.3 in the Cd between the habit and the non-habit tree shrews. (**B**,**F**,**H**) No differences were observed in the membrane protein level of D1Rs, Ca_v_1.2 and Ca_v_1.3 in the Cd between the habit and the non-habit tree shrews. (**D**) The membrane protein level of D2Rs in the Put was lower in the habit tree shrews than in the non-habit animals. * *p* < 0.05, habit group vs. non-habit group. The data were expressed as the means ± SEM and analyzed with the *t*-test, habit group *n* = 4, non-habit group *n* = 4.

To further evaluate the changes of these molecules between the well-established sucrose habitual behavior tree shrews and the non-habitual animals, we observed no differences between the habitual group and the non-habitual group in the total protein levels of Ca_v_1.2, Ca_v_1.3, D1Rs or D2Rs in the Put (Figure 5A,C,E,G). Then, the statistical data showed no difference between the habit tree shrews and the non-habit tree shrews in the membrane protein levels of Ca_v_1.2, Ca_v_1.3, or D1Rs in the Put (Figure 5B,D,H), but the membrane protein levels of D2Rs in the Put were significantly lower in the well-established habitual sucrose-seeking tree shrews than in the non-habit tree shrews (*t*-test, *t* = 2.512, *p* = 0.046) (Figure 5F). Our results showed that the membrane protein levels of D2R of the Put decreased in the habit tree shrews compared with the non-habit animals.

**The protein levels of D1R, D2R, Ca_v_1.2 and Ca_v_1.3 were no different in the Cd of sucrose-habitual tree shrews.**

**Figure 6:**
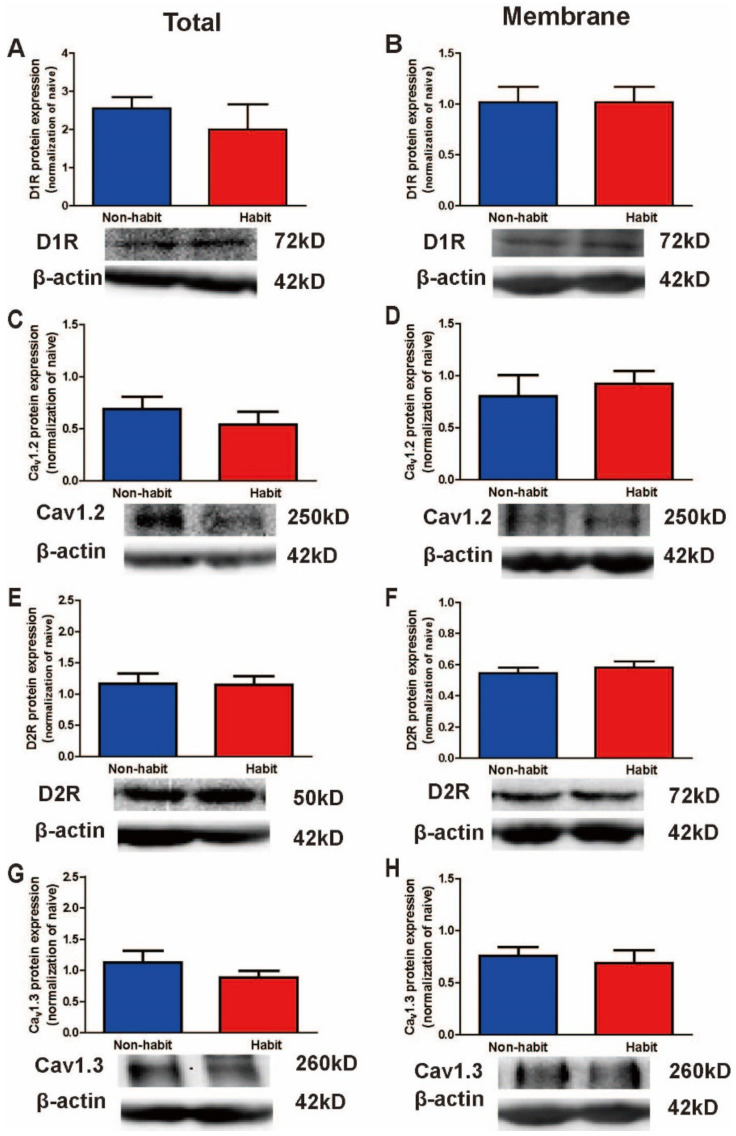
**The total and membrane protein levels of D1Rs vs. D2Rs, Ca_v_1.2 vs. Ca_v_1.3 in the Cd.** (**A**,**C**,**E**,**G**) No differences were observed in the total protein level of D1Rs, D2Rs, Ca_v_1.2, and Ca_v_1.3 in the Cd between the habit and the non-habit tree shrews. (**B**,**D**,**F**,**H**) No differences were observed in the membrane protein level of D1Rs, D2Rs, Ca_v_1.2, and Ca_v_1.3 in the Cd between the habit and the non-habit tree shrews.

We detected the total and membrane protein levels of Ca_v_1.2, Ca_v_1.3, D1Rs, and D2Rs in the Cd in the habit group and non-habit group with the method of western blot. We observed no differences between the habit tree shrews and the non-habit animals in both total and membrane protein levels of these molecules in the Cd (Figure 6).

The data were expressed as the means ± SEM and analyzed with the *t*-test, habit group *n* = 4, non-habit group *n* = 4.

## 4. Discussion

Our current study showed that both food and cocaine-seeking habits displayed insensitivity to the devaluation tests. Only forty percent of tree shrews exhibited habitual cocaine-seeking behavior, whereas the rate increased to fifty percent in the sucrose group. However, as the sample size was relatively small, this observation needs to be further validated. Moreover, we found differential alterations of dopamine receptors and L-type calcium channel subtypes between cocaine-seeking habit and sucrose-seeking habit tree shrews compared with non-habit tree shrews. Burgeoning evidence points to a maladaptive habit system underlying the behavioral manifestation of addiction. For instance, non-contingent exposure to cocaine or amphetamine expedites the formation of habitual behavior reinforced by sucrose [25,26]. In humans, addicts also represent over-reliance upon the habit system [2]. The above findings highlight the importance of comparative studies between “normal” habits and maladaptive habits in addiction.

The Put plays a key role in mediating habitual behavior. Indeed, pharmacological blockade of the Put impaired the expression of habitual behavior, but did not influence the acquisition. One of the main regulators of the Put activity is DA receptor system. There are two subtypes of DA receptors: D1-like subtype couples to the G protein Gs, which activates adenylyl cyclase (AC) and recruits Ca_v_1.2-dependent signaling, further enhancing the neural activity; while the D2-like subfamily instead inhibits AC, recruits Ca_v_1.3 signaling, and subsides neuronal excitation. Based on that, we tested the protein levels of DA receptor subtypes and LTCCs subtypes between the habit and non-habit groups after cocaine and sucrose SA training, respectively. We found that, compared with the non-habitual cocaine-seeking group, there was higher expression of both D1Rs and Ca_v_1.2, and lower expression of both D2Rs and Ca_v_1.3 in the Put in the habitual cocaine-seeking group. However, conversely, we only found decreased D2Rs in the Put in the habitual sucrose-seeking group. These results suggest that the up-regulation of D1Rs-Ca_v_1.2 signaling and the down-regulation of D2Rs-Ca_v_1.3 signaling may cause maladaptive hyperactivity of the Put, which likely underlies maladaptive elements of cocaine-seeking habit. 

Furthermore, in the striatum, two fundamental neural circuits are constituted by specified medium-sized spiny neurons (MSNs), each expressing a distinct type of DA receptor. One circuit is the direct pathway, predominantly expressing dopamine D1 receptors (D1Rs). The other is the indirect pathway, primarily expressing dopamine D2 receptors (D2Rs). Therefore, our results also suggest that the imbalanced activity between direct MSNs (dMSNs) and indirect MSNs (iMSNs), which was evaluated by D1Rs and D2Rs, causes it to be difficult to shift from goal-directed to habitual behavior when the environment or reward value changes.

The method that we used to establish cocaine-seeking habit was two days of FR1 followed by six days of VI schedules. Similar to the rats [27,28], forty percent of tree shrews showed habitual cocaine-seeking. No significant difference in the number of valid nose pokes was observed in the last three sessions of FR1 between the habit and non-habit groups. These results indicated that both groups performed the well-established SA training and showed no difference in learning ability. Moreover, from the beginning of the VI training, the habit animals were more insensitive to the changeable delayed reward time than those in the non-habit group. In addition, the total doses of cocaine were not significantly different between these two groups. Therefore, the causes for the establishment of habitual drug-seeking behavior included not only the cocaine itself but also the vulnerability of these tree shrews. For instance, studies have shown that animals with lower D2R expression itself are more impulsive [29,30], which might act as an intrinsic character of vulnerability to habitual drug-seeking. 

Evidence has demonstrated that the expression of habitual drug-seeking behavior depends on the activation of the dorsal striatum (DS) as regulated by dopaminergic input from the substantia nigra (SN) [31]. Indeed, dopaminergic nuclei are becoming a target for potential treatment [32], and recent studies have further found that blocking DA receptors in the striatum inhibits well-established drug-seeking behavior [21]. Our results found that the protein level of D1R was higher, whereas the protein level of D2Rs was lower in the Put in habitual cocaine-seeking tree shrews compared with non-habitual animals, indicating that the Put might be highly active via upregulating D1Rs signaling and downregulating D2Rs signaling. Furthermore, the increase of D1Rs and the decrease of D2Rs in our present results also imply the activation of dMSNs and inactivation of iMSNs, and it has been shown that these two types of neurons usually compete for action control. Based on these results, the enhanced S-R action may be induced by D1Rs increase and the activation of dMSNs, resulting in the habitual drug-seeking. 

More specifically, activation of iMSNs mainly supports the A-O action strategies [11,33,34], while dMSNs activation supports the S-R action strategies [35,36,37]. In addition, studies have shown that the activation of D1Rs in the dMSNs enhances neural excitability via protein kinase A (PKA) signaling and is essential for the expression of long-term plasticity in the dorsal striatum. In contrast, the activation of D2Rs worked in the opposite way, and is necessary for expressing the long-term depression [8]. Therefore, it is possible that the upregulated D1Rs and downregulated D2Rs make dMSNs and iMSNs more readily activated by dopamine (DA), leading to habitual drug-seeking behavior. It was consistent with other results in that both pathways participated in reward-seeking behavior in the contingency degradation (CD) session, while iMSNs were activated earlier than dMSNs [5,38]. Furthermore, in contrast to cocaine, habitual sucrose-seeking behavior was only accompanied with the decrease of D2Rs level, and there was no change in D1R level related to the habitual sucrose seeking. This result was similar to other studies in that the downregulation of D2Rs was found in natural reward (such as delicious food) habitual behavior [39,40]. In addition, both the food and cocaine groups showed lower D2R expression in habitual animals than non-habitual animals, but only the habitual cocaine-seeking animals showed higher D1R expression in the Put. These results suggested the upregulation of D1R was specific to the habitual drug-seeking behavior. It raised the possibility that an abnormal increase of D1Rs in the Put might be necessary for habitual drug-seeking and difficulty to switch actions and return to goal-directed strategies even when faced with serious negative consequences. 

In our present study, we also evaluated the changes of DA receptors in the Cd of tree shrews, which is the same as the dorsomedial striatum (DMS) in the rodents. Different from the Put, there was no adaptive change of D1Rs and D2Rs in the Cd after the expression of habitual drug- or sucrose-seeking. It was consistent with studies showing that blockade of DA receptors in the DMS only impaired habitual drug-seeking at the early stage but had no effect on the established habitual behavior [1,41]. Lesions of the posterior DMS abolished the sensitivity of rats’ instrumental performance to outcome devaluation, implying that DMS played an essential role in the goal-directed action [40]. Moreover, intracranial self-stimulation of dMSNs in the DMS leads to reinforcement of actions, while the same manipulation in the iMSNs leads to avoidance of actions [42]. Therefore, the activation of DMS might be necessary for the formation or development of instrumental lever-pressing associated learning through guiding the action according to goal-directed strategies. Nevertheless, it also raised another possibility that the DMS might decrease activity during the expression of habitual drug-seeking, which was reported in our previous study in rats [13]. However, in the Cd of tree shrews, we did not detect changes in DA receptor signaling after cue exposure in the well-established habitual cocaine or sucrose-seeking animals. Some factors, such as species of animals, location of the focus brain regions, or the training procedures, can explain these differences. For example, for tree shrews, the division of function related to the execution of behavior strategies might be more specialized, equal to saying that the Put activation might be enough to express the well-established habitual behavior.

Our results showed that the protein level of Ca_v_1.2 in the Put exhibited the same dynamic tendency as D1Rs, and the protein level of Ca_v_1.3 in the Put showed the same tendency as D2Rs. D1Rs influence Ca_v_1.2 by activating the PKA pathway [8,43], while D2Rs can regulate Ca_v_1.3 through a calcineurin-dependent mechanism [8], indicating that DA receptor system works closely with Ca_v_1.2 and Ca_v_1.3. Moreover, D1Rs and D2Rs can exert their effects on the same process of drug addiction by modulating Ca_v_1.2 and Ca_v_1.3, respectively [44]. In the expression of cocaine sensitization, Ca_v_1.2 works as one of the critical modulators, regulated by the activation of D1Rs, phosphorylates GluA1 of a-amino-3-hydroxy-5-methyl- 4-isoxazole-propionic acid receptor (AMPAR) at the Ser831 site, leading to the LTP [14,45]. Conversely, the decreased D2Rs stimulated Ca_v_1.3 in the DS to suppress its downstream activation, and reduced phosphorylation at GluA1 at the Ser845 site, resulting in the LTD [14,45]. This evidence implies that the upregulation of D1Rs signaling by moderating the activation of Ca_v_1.2 might cause enhanced synaptic efficacy in striatonigral neurons, which supports S-R action. Meanwhile, the downregulation of D2Rs pathway by mediating the activation of Ca_v_1.3 might induce depressed synaptic efficacy in striatopallidal neurons, which supports goal-directed action, finally leading to the establishment and expression of habitual cocaine-seeking behavior. Furthermore, we also evaluated the protein level of LTCCs subtypes in the sucrose group, and there were no changes of Ca_v_1.2 and Ca_v_1.3 in either the Put or the Cd between habit and non-habit groups. These results indicated that the variation of LTCCs, monitored by the DA system, might be the specific molecular mechanism involved in drug-related habitual behavior.

## 5. Conclusions

Using the VI training schedule, we successfully established a tree shrews model of habitual cocaine-seeking behavior, and also established habitual sucrose-seeking behavior to investigate the distinct molecular mechanisms between addictive drugs and the nature reward. Furthermore, we found that the protein expression of both D1Rs and Ca_v_1.2 were higher and that the protein expression of D2Rs and Ca_v_1.3 were lower in the Put in habitual cocaine-seeking tree shrews than in the non-habitual group. In contrast, habitual sucrose-seeking animals were only related to the decrease of D2Rs in the Put. It implied that an abnormal increase of D1Rs in the Put might be necessary for habitual drug-seeking behavior.

## Data Availability

The data that support the findings of this study are available from the corresponding author upon reasonable request.
